# Extraventricular neurocytoma in pediatric populations: A case report and review of the literature

**DOI:** 10.3892/ol.2013.1583

**Published:** 2013-09-12

**Authors:** LIN HAN, HONGQUAN NIU, JUNWEN WANG, FENG WAN, KAI SHU, CHANGSHU KE, TING LEI

**Affiliations:** 1 Department of Neurosurgery, Tongji Hospital, Tongji Medical College, Huazhong University of Science and Technology, Wuhan, Hubei 430030, P.R. China; 2 Department of Neurosurgery, Center Hospital of Wuhan City, Wuhan, Hubei 430014, P.R. China; 3 Department of Pathology, Tongji Hospital, Huazhong University of Science and Technology, Wuhan, Hubei 430030, P.R. China

**Keywords:** extraventricular neurocytoma, pediatrics

## Abstract

Extraventricular neurocytomas (EVNs) are rare neuronal tumors included in the definition of neoplasms in the 2007 World Health Organization classification of tumors of the central nervous system. Although a small case series of EVNs in adults has been previously reported, EVNs in pediatric populations are extremely rare. The current case report presents the clinicopathological features of an EVN in a 2-year-old female who presented with nausea and vomiting that had lasted for five days. In addition, an analysis of the imaging features, histology, treatment and prognosis of these reported rare lesions is presented. Immunohistochemically, EVNs are characterized by the robust expression of synaptophysin, but with a lack of oligodendrocyte transcription factor 2, isocitrate dehydrogenase enzyme isoform 1 (IDH1) R132/IDH2 R172 mutations and p53 immunoexpression. The treatment for EVNs in pediatric and adult populations is gross total resection, with post-operative radiation reserved for subtotal resection or recurrent disease. In addition, drop metastasis must be carefully avoided.

## Introduction

Neurocytomas are rare tumors hypothesized to originate from bipotent progenitor cells, with the potential for neuronal and glial differentiation (1,2). Extraventricular neurocytomas (EVNs) are rare neuronal tumors included in the definition of neoplasms in the 2007 World Health Organization (WHO) classification of tumors of the central nervous system (3). EVNs tend to be large, well-circumscribed lesions located in the cerebral hemispheres that are commonly identified in the frontal and parietal lobes. However, EVNs have been located in the thalamus, cerebellum, pineal region and even in the spinal cord (4–6). Unlike the usual intraventricular central neurocytomas (CN), typical EVNs exhibit a wide spectrum of morphologies, including the growth of monotonous neurocytes in sheets, clusters, ribbons or rosettes and neurophils dispersed in broad zones (7). Although EVNs exhibit histologically uniform round cells, i.e. a clear cell morphology with neuronal differentiation, a clear cell morphology is also a classic feature of oligodendroglioma. Thus, the morphological overlap of these tumors often creates diagnostic difficulties (8,9). Moreover, increasing evidence of oligodendrogliomas with neuronal or neurocytic differentiation makes the distinction of EVNs increasingly difficult despite various immunohistochemistry methods of examination (10). Familiarity with the histopathological features of neurocytoma is a necessity for pathologists and surgeons in order to avoid such a misdiagnosis and to provide optimal therapy. While a small case series of EVNs in adults has been previously reported, EVNs in the pediatric population are extremely rare (11). To aid the clarification of the spectrum of such lesions and their biological behavior in the pediatric population, the present case report presents the clinicopathological features of an EVN in a 2-year-old female. The patient was admitted to hospital with nausea and vomiting that had lasted for five days and a large right frontal lobe mass that was later determined to be an EVN. In addition, the current study presents an analysis of the imaging features, histology, treatment and prognosis of these reported rare lesions. Written informed consent was obtained from the patient.

## Case report

**Figure 1:**
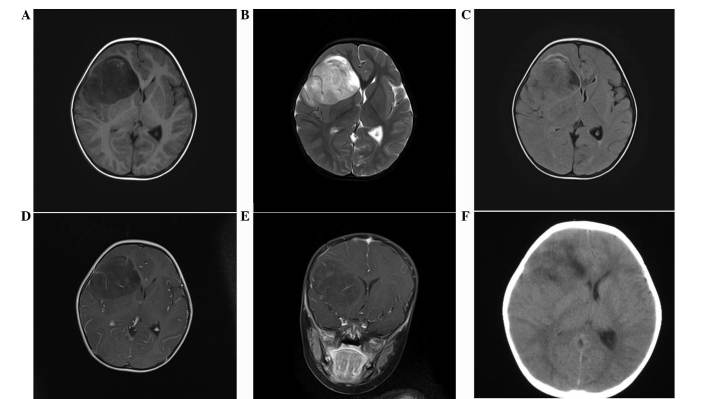
EVN identified in the right frontal cortex and subcortical white matter of a 2-year-old female. (A) T1-weighted image, (B) T2-weighted image, (C) FLAIR image and (D) axial and (E) coronal post-contrast T1-weighted image showing a moderate-to-marked diffuse and enhanced right frontal lobe lesion involving mainly the cortex and subcortical white matter. Peritumoral edema, cysts, hemorrhages and calcification were not observed. (F) CT scan revealed a large, microcystic and non-calcified right frontal lobe mass. EVN, extraventricular neurocytoma; FLAIR, fluid-attenuated inversion recovery.

A previously healthy 2-year-old female was admitted to Tongji Hospital (Wuhan, China) with nausea and vomiting that had lasted for five days. Upon physical examination, a bilateral papilloedema was noted, but no other neurological deficits were observed. A non-contrast head computed tomography (CT) scan showed a large, microcystic and non-calcified right frontal lobe mass with an ~10 mm right-to-left shift and effacement of the frontal horn of the right lateral ventricle (Fig. 1). There was a small amount of vasogenic edema surrounding the lesion, particularly on its medial aspect (Fig. 1).

Treatment with 20% mannitol (50 ml) and dexamethasone (1 mh per 6 h) was initiated for the increased intracranial pressure and cerebral edema. Magnetic resonance imaging (MRI) revealed a T1 hypointense lesion with solid and microcystic components and mild perilesional edema in the right frontal lobe. Post-contrast, the lesion showed mild striped enhancement, while restricted diffusion was observed in the solid component (Fig. 1). There was a mass effect on the ipsilateral lateral ventricle, a midline shift of ~10 mm of the right frontal cortex and subfalcine herniation (Fig. 1). On T2-weighted MRI, the lesion was heterogeneously hyperintense and mild peritumoral edema was identified in the frontal and temporal lobe (Fig. 1).

**Figure 2:**
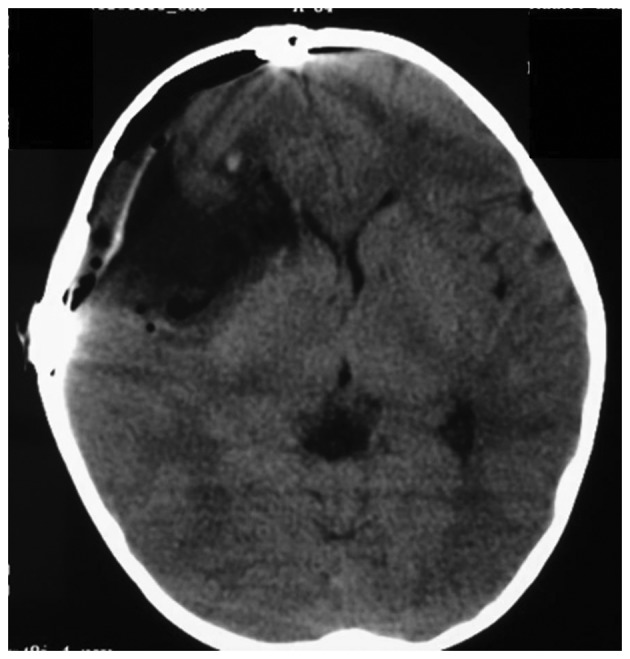
A CT scan was performed following the initial resection and showed gross total resection of the tumor. CT, computed tomography.

**Figure 3:**
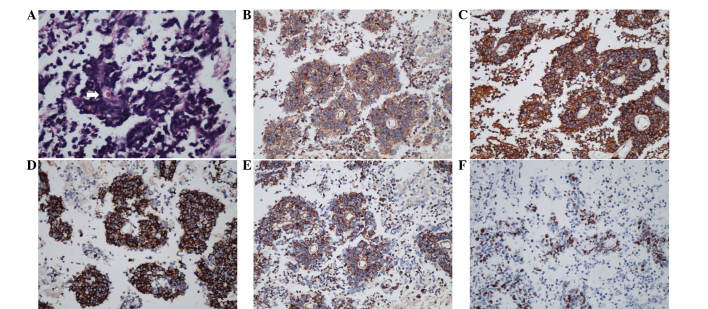
Histopathology of the lesion resected during the craniotomy of a 2-year-old female. (A) Hematoxylin and eosin staining indicates a tumor of moderate cellularity with vascular proliferation, as indicated by the arrow (magnification, ×400). Immunohistochemistry demonstrating neoplastic cells exhibiting strong, diffuse immunoreactivity for (B) CD99 and (C) MAP2, immunoreactivity for (D) nestin and (E) vimentin and a MIB-1 (Ki-67) labeling index (LI)of 20% (magnification, ×200). MAP2, microtubule-associated protein 2.

The patient underwent a right pterional craniotomy for tumor resection. The tumor was not adherent to the overlying parenchyma and there was not an excessive amount of vascularity. The patient exhibited no neurological deficits post-operatively, and post-operative CT showed gross total resection (GTR) of the lesion (Fig. 2). Histologically, the tumor exhibited uniform small round cells with regular nuclear morphology (Fig. 3) and areas of tumor apoplexy. Immunohistochemically, the tumor cells showed perinuclear positivity for synaptophysin (Syn) and focal positivity for oligodendrocyte transcription factor 2 (Olig2). In addition, strong immunopositivity for nestin, microtubule-associated protein 2, vimentin and CD99 was noted. There was no positive staining for glial fibrillary acidic protein (GFAP), epithelial membrane antigen, neurofilaments and NeuN. The MIB-1 (Ki-67) labeling index (LI) was 20% (Fig. 3). The concluding histological diagnosis was atypical EVN (WHO grade III).

**Figure 4:**
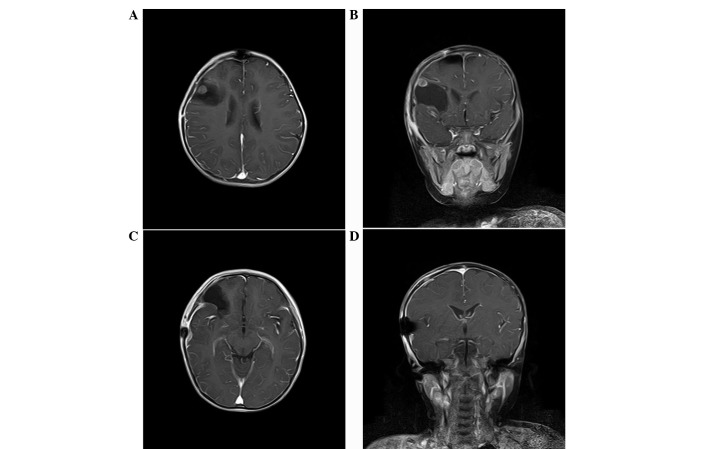
MRI scan with gadolinium diethylenetriamine pentaacetic acid administration was performed one month after the initial resection. (A) A solid node in the initial surgery field was identified and (B) a coronal image was captured. (C and D) Enhancement of the other intradural tumor node was identified at the posterior edge of the bone window. MRI, magnetic resonance imaging.

**Figure 5:**
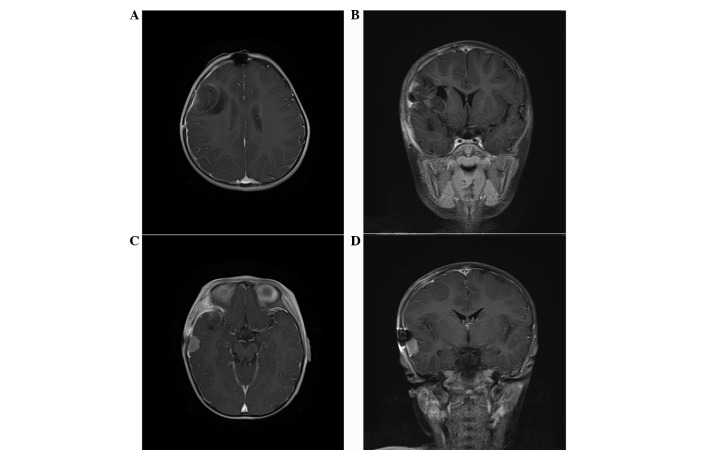
MRI scan with enhancement performed 10 weeks after the initial resection, showing an enlargement of the two tumor nodes identified six weeks previously. (A and C) Axial and (B and D) coronal images were captured. Notably, the tumor node located in the initial surgery field showed mild enhancement, but the other tumor node located in the area posterior-inferior to the initial surgery field showed significant enhancement. MRI, magnetic resonance imaging.

Following surgery, adjuvant radiotherapy was refused by the parents due to the patient’s young age. The patient recovered well, remained neurologically intact and was discharged from hospital 8 days after the surgery. However, at the 4-week follow-up, the patient had developed a new-onset of generalized tonic-clonic seizure episodes. Treatment with levetiracetam (200 mg/day) was initiated for seizure prophylaxis. Complete spinal imaging was performed using MRI, with no evidence of long distance subarachnoid dissemination of the tumor being identified. However, cerebral MRI revealed two small solid nodules along the frontotemporal dura. One recurred in the initial operative area and the other in the area posterior-inferior to the initial operative area (Fig. 4). Adjuvant radiotherapy and chemotherapy were refused by the patient’s parents again. Ten weeks after the resection, the patient developed a recurrence with nausea and vomiting, and MRI identified enlargement of the two solid nodes (Fig. 5). A craniotomy and tumor resection was refused. Following one week of conservative therapy, the patient succumbed to EVN.

## Discussion

To date, limited information on EVNs in the pediatric population has been reported, and the literature is composed almost exclusively of case reports. Tumors are classified into typical or atypical categories, and atypical histological criteria are designated by an MIB-1 LI of >3% or features consistent with higher grade tumors (12). The current case report presents a rare example of an atypical EVN in a 2-year-old female presenting with new-onset nausea and vomiting. This case illustrates the fact that atypical EVNs must be included as a possibility for a non-calcified and microcystic parenchymal lesion in the pediatric population. A literature review via the PubMed database was performed using the keywords, ‘extraventricular’, ‘neurocytoma’, ‘children’ and ‘pediatrics’, alone and in combination. Subsequently, the references in these studies were investigated for additional reports. The search identified 28 studies reporting on 44 patients with EVNs that met the inclusion criteria (6–9,11,13–35). In addition, three other reported pediatric cases were identified in the Chinese literature (36–38). For each study, the age, tumor location, treatment modality, histopathological report, follow-up duration and recurrence data were extracted for individual patients. Overall, 47 pediatric cases were identified up to January, 2013. The tumor location is summarized in Table I, and the demographic data and clinical characteristics for patients with atypical EVNs are summarized in Table II.

The collective case studies on EVNs indicated a slight male predominance (29 males/18 females). Seizure activity was the most common presenting symptom (15/47 patients), followed by headaches (11/47 patients). These tumors may exhibit atypical features consistent with aggressive clinical behavior. The tumors were often cystic (16/47 patients) with frequent calcifications and mild peritumoral edema. Garber and Brockmeyer reported that this may aid the radiographical identification of EVNs from oligodendrogliomas (19). By contrast, Yi *et al* reported that the majority of the cases (90%) demonstrated no peritumoral edema (35). No significant peritumoral edema was identified in the patient of the present case report. Consistent with the results of the present case, the majority of reported EVNs are hypointense on T1-weighted MRI and hyperintense on T2-weighted MRI, with heterogeneous enhancement following the administration of a contrast agent (35). Therefore, EVN clinically presents as a diagnostic challenge. The primary differential diagnosis of EVN is oligodendroglioma with neurocytic differentiation, oligoastrocytoma with neurocytic differentiation, ganglioglioma, DNT and pineal parenchymal tumor of intermediate differentiation (7).

In 1997, Giangaspero *et al* reported a series of 11 patients diagnosed with EVNs (11). Three of the patients were children with typical EVNs. Although one patient succumbed to EVN following subtotal resection (STR) of a tumor located in the hypothalamus, two underwent GTR with no evidence of disease after 29 and 31 months, respectively. Of the total 85 EVNs previously reported in the literature, 27% were atypical (23/85 patients) (39). Atypical tumors exhibit 2–3 times the recurrence risk of typical EVNs, as well as recurring at a much earlier time post-treatment. A Kaplan-Meier analysis demonstrated significant differences between patients with typical and atypical tumors and post-primary treatment 5-year recurrence rates (36 and 68%, respectively; P<0.001) and 5-year mortality rates (4 and 44%, respectively; P<0.001) (39). However, complete resection may not be possible due to the eloquence of surrounding structures or the invasion of the surrounding periventricular parenchyma. This may lead to a poorer prognosis in EVNs. In cases of typical EVNs, overall recurrence rates have been recorded as 5% following GTR and 32% following STR (P<0.05; χ^2^ test). In cases of incomplete resection, radiotherapy offers local control, but does not appear to affect overall survival (11). STR with adjuvant radiotherapy was associated with a 17% recurrence rate, which was not significantly different when compared with STR alone. Disease progression was observed in 20% of patients following subtotal resection at a mean time of 30 months (40). As observed in the present patient, adjuvant radiotherapy is advised in cases following subtotal resection/biopsy. However, the value of radiation therapy as adjunct therapy is debatable, as the majority of clinicians agree that radiation therapy must be provided in cases of incomplete resection or atypical histology (8,41). In general, the literature on EVNs supports a total resection as an optimistic treatment and the mandatory close follow-up of cases with subtotal removal or aggressive histological features. Post-operative chemotherapy and radiation are reserved for patients that exhibit STR or recurrent disease.

EVN frequently demonstrates specific patently neuronal features, including rosette and neuropil formation. This is in addition to immune expression of multiple markers of neuronal differentiation, particularly Syn in a diffuse manner (42). GFAP, Syn and NeuN are reliable markers for glial and neuronal differentiation. Oligodendrogliomas may possess neuronal differentiation and exhibit positive immunostaining for markers of a neuronal tumor. Olig2 has been identified as a transcription factor that regulates oligodendroglial development and has been reported to be useful in determining the diagnosis of oligodendroglioma (43,44). In addition, central neurocytomas have been reported to be immunohistochemically negative for Olig2 (44). Therefore, if tumor cells are positive for Olig2, it is inappropriate to consider the tumor as a neurocytoma. Despite immunostaining, it is difficult to differentiate between oligodendrogliomas and EVNs. Studies analyzing the genetic abnormalities of EVN have been seriously limited. The codeletion of 1p19q has been described in a minority of EVNs (45). Rodriguez *et al* reported that the 1p19q codeletion occurred in 24% of EVNs and that, as with oligodendroglial tumors, it was mediated in the majority of cases by t(1;19)(42). Commonly, the 1p19q codeletion predicts a more favorable prognosis and an improved response to treatment in oligodendrogliomas, however, this is not the case in EVNs (42). In the present literature review, the codeletion was identified in 5/23 cases and all were adult patients. Of the five patients, three exhibited recurrence and two succumbed to EVN within 5 years of the initial tumor resection. Four patients exhibited features of histological atypia by pathological analysis, including necrosis and vascular hypertrophy. This indicated that in EVNs, the presence of a 1p19q codeletion, although rare, may imply aggressive clinical behavior and a poorer outcome (46). Mutation of the isocitrate dehydrogenase enzyme isoform 1 (IDH1 R132) and 2 (IDH2 R172) is associated with a large proportion of diffuse astrocytic and oligodendroglial tumors. A recurrent mutation affecting codon 132 of the IDH1 gene, located on chromosome 2q33, is used for differentiating oligodendroglioma-like tumors from others (47). High numbers of IDH1 point mutations at codon 132 were observed in oligodendrogliomas and oligoastrocytomas. All observed neurocytomas (central, n=35; extraventricular, n=4) were negative for mutated IDH1 protein. In an additional series of seven EVNs, IDH1 immunostaining was negative for all cases, and IDH1 R132 and IDH2 R172 direct sequencing revealed wild types in all cases (7). It was identified that the absence of IDH1 expression functions as a powerful diagnostic indicator for EVN-mimicking gliomas. A recent study by Yi *et al* also reported observations of a series of IDH1 mutation-negative EVNs, as determined by MRI (35). Therefore, it may be hypothesized that the absence of the gene mutation of IDH1 represents a prerequisite for EVN diagnosis. In addition, the absence of p53 immunoexpression, MGMT promoter methylation and a low frequency of EGFR gene amplification are significant features of EVNs used to differentiate between astrocytic and oligodendroglial tumors (7).

Neurocytomas are benign, slow-growing central nervous system tumors of neuroglial origin (46). These tumors represent 0.25–0.5% of all intracranial tumors in adults and an even smaller proportion of pediatric CNS tumors. In 2001, Brat *et al* examined 33 pediatric and adult patients with EVNs (8). Within this study group, 14 patients underwent GTR, whilst 19 patients underwent STR or biopsy. Of the 14 patients treated with GTR, no tumor was observed to recur during the mean follow-up time of 29 months. Among the 19 patients who underwent STR, three succumbed to EVN and ten exhibited recurrence, with a median time to recurrence of 17 months. Patients with tumor recurrence tended to exhibit a higher proportion of histological atypia. Recurrence and mortality rates for typical CNs are not dissimilar from those reported for typical EVNs, with 28% recurrence and 5% mortality in typical CNs compared with 36% recurrence and 4% mortality in typical EVNs (48). Atypical features appear to lead to higher rates of recurrence and mortality in CNs and EVNs, with 40% recurrence and 20% mortality in atypical CNs and 68% recurrence and 44% mortality in atypical EVNs (49). The extent of the resection appears to be a significant prognostic indicator, as no completely resected EVNs recurred, whereas >50% of patients who underwent STR experienced recurrence. In addition, 12 cases of CN with craniospinal dissemination following operative intervention have been reported in the literature to date, indicating that an aggressive, atypical form of this tumor may exist (50–52). Stapleton *et al* recently reported a rare case in the pediatric population of a diffuse CN with craniospinal dissemination that was identified at the time of the initial diagnosis by the immunohistochemical results of an elevated Ki-67 proliferation index (53). To date, there have been five reported cases of EVN with craniospinal dissemination (8,30,49,54–56), including four males (7, 24, 48 and 75 years old) and one female (71 years old). Two of the patients showed drop dural metastasis and one tumor was initially located in the sellar region and was associated with multiple remote disseminations in the spinal cord and drop metastasis in the frontal cranial base in the route of the initial surgery (54). The other tumor was initially located in the left occipital-parietal lobe and was associated with drop metastasis along the frontotemporal dura with an intratumoral hemorrhage outside the field of the initial surgery (56). The initial tumor of the patient in the present case report was located in the right frontal lobe. Four weeks after the initial surgery, cerebral MRI revealed two small solid nodules along the frontotemporal dura, one located in the field of surgery and the other located at the trailing edge of the bone window. All tumors were considered atypical with MIB-1 LI ranging between 4 and 30% (Table III). GTR was achieved in 4 of the 5 patients at the initial therapy, and additional radiation therapy was delivered to one of the cases. However, the tumors recurred several months later or disseminated into the craniospinal cavities. A recent study by Kane *et al* observed that GTR and STR with adjuvant radiotherapy appeared to offer improved post-treatment tumor control rates for atypical EVNs (39). An additional recent study described the use of GTR and STR with high dose chemotherapy, autologous stem cell rescue and adjuvant therapy in a 9-month-old female with recurrent atypical CN and leptomeningeal spread. The patient exhibited a complete response to therapy and remained disease-free at 4 years of age, until a recurrence 6 months later (16). These observations indicate that, for atypical cases, long-term follow-up is required even when complete remission has been achieved and novel treatment strategies, including radiotherapy, chemotherapy and/or molecular targeted therapies, have been used. The use of intensive chemotherapy followed by autologous stem cell rescue for atypical neurocytoma may be considered as an adjunct to surgical therapy in young patients with atypical neurocytoma not amenable to radiation therapy (16).

EVNs are rare intraparenchymal lesions that must be included in the differential diagnosis of a cerebral mass in children. Although the imaging features of EVNs are variable, they are usually cortically-based hemispheric lesions with variable contrast enhancement and a cystic component, but they do not show peritumoral edema. The frontal and temporal lobes are most commonly involved. Immunohistochemically, EVNs are characterized by the robust expression of Syn, but lack Olig2, IDH1 R132/IDH2 R172 and p53 immunoexpression. The treatment for EVNs in the pediatric and adult populations is GTR, with post-operative radiation reserved for STR or recurrent disease. In addition, drop metastasis must be carefully avoided. Recurrence and mortality rates remain high in atypical EVN cases despite adjuvant radiation therapy. Therefore, future studies must focus on determining successful chemotherapy regimens and identifying novel molecular markers for targeted adjuvant therapies.

## Figures and Tables

**Table I:** Summary of the frequency of EVNs derived from 35 studies that met the inclusion criteria.

Reported location	Lesions, n (%)
Frontal lobe	16 (34)
Temporal lobe	6 (13)
Parietal lobe	5 (11)
Occipital lobe	1 (2)
Frontoparietal lobe	2 (4)
Frontotemporal lobe	1 (2)
Parietotemporal lobe	1 (2)
Thalamus	2 (4)
Cerebellum	4 (9)
Vermis	2 (4)
Spinal	6 (13)
Mesencephalon	1 (2)
Total	47 (100)

EVNs, extraventricular neurocytomas.

**Table II:** Summary of demographic and lesion-associated data in cases of atypical EVNs reported in the previous literature.

First author (ref.)	Age, years/gender	Tumor location	Resection	Calcification	Pathology	MIB-1, %	Adjuvant therapy	Recurrence time	Follow-up, months/outcome
Ghosal *et al*(20)	9/M	Frontoparietal	ND	Yes	Syn (f), GFAP (f) and NF (++)	10.0	ND	ND	ND
Singh *et al*(31)	8/M	Spinal D2-8	STR	No	Syn (+), NSE (+), GFAP (−), EMA(−) and NF (−)	13.0	No	ND	3/normal
Raja *et al*(30)	7/M	Occipital	GTR	Yes	Syn (+), GFAP (±) and NeuN (±)	4.0	Chemotherapy	3 months	6/cerebral spinal dissemination
Mpairamidis *et al*(25)	3/F	Parietotemporal	GTR	No	Syn (+), NeuN (+), NF(−), β-tubulin III (−), MBP (−) and GFAP (±)	5.0	RT	ND	12/normal
Choi *et al*(18)	8/M	Frontal	STR	Yes	Syn (++)	ND	γ-knife	15 years	12/second surgery normal
Myung *et al*(7)	9/M	Frontal	STR	Yes	Syn (+), GFAP (+), Olig2 (±), IDH1(−), p53 (−) and NeuN (−)	16.8	ND	13.9 and 17.2 years	ND
Agarwal *et al*(13)	16/M	Spinal	STR	ND	Syn (+), GFAP (−), NeuN (+) and NF (−)	22.0	RT	ND	6/normal
Buchbinder *et al*(16)	1/F	Frontal	GTR	No	Syn (+) and GFAP (−)	10.0	Chemotherapy	2 months	46/cerebral spinal dissemination

Pathology grades: f, focal area positive; −, negative; +, positive; ++, strong positive; ±, a number of cells positive. GTR, gross total resection; STR, subtotal resection; RT, radiation therapy; GFAP, glial fibrillary acid protein; Olig2, oligodendrocyte transcription factor 2; MBP, myelin basic protein; NF, neurofilament; NSE, neuron-specific enolase; Syn, synaptophysin; IDH1, isocitrate dehydrogenase enzyme isoform 1; NeuN, neuronal nuclear antigen; EVN, extraventricular neurocytoma; EMA, epithelial membrane antigen; M, male; F, female; ND, no description.

**Table III:** Extraventricular neurocytoma with craniospinal dissemination.

First author (ref.)	Age, years/gender	Tumor site	Initial therapy	MIB-1, %	Follow-up, months/outcome
Brat *et al*(8)	71/F	Cerebrum	Biopsy and RT	ND	18/Succumbed to the disease
Sharma *et al*(55)	24/M	Spine C5-T1	GTR	9	14/Cerebellar metastasis
Raja *et al*(30)	7/M	Cerebrum	GTR	4	6/Spinal canal dissemination suspected
Wang *et al*(56)	75/M	Cerebrum	GTR and RT	>30	7/Dural metastasis
Kawaji *et al*(54)	48/M	Sellar region	PR and RT	3	6/Spinal canal dissemination and metastasis in frontal cranial base
Present case	3/F	Cerebrum	GTR	20	1/Dural metastasis

M, male; F, female; GTR, gross total resection; PR, partial resection; RT, radiotherapy; ND, not described; EVN, extraventricular neurocytoma.
